# Michel’s Anatomy of the Bullet Track and Cancroid of the Lower-Lip

**Published:** 1875-09

**Authors:** 


ANATOMY OF THE BULLET-TRACK, and Cicatrices of the Wounds of Entrance and Exit. By Middleton Michel, M.D., Professor of Physiology in Medical College of South Carolina. Charleston: 1875. Pamphlet, pp. 14.

---

CANCROID OR EPITHELIOMA OF THE LOWER-LIP; Modified Operation for its Removal; Cure. By Middleton Michel, M.D., etc. Charleston: 1875. Pamphlet, pp. 6.

Dr. Michel proposes to revive an old method, by which the mucous lining of the lip and the integument are preserved as far as possible, instead of sacrificing the whole by the usual V-shaped incision.

